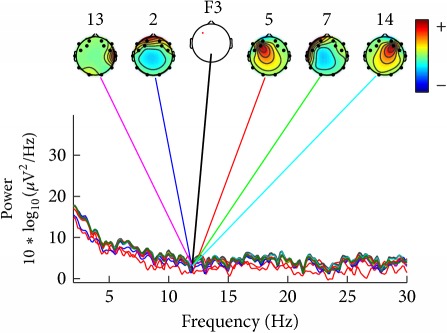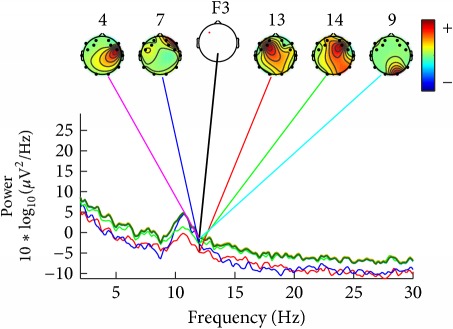# Auris System: Providing Vibrotactile Feedback for Hearing Impaired Population

**DOI:** 10.1155/2017/2181380

**Published:** 2017-09-12

**Authors:** Felipe Alves Araujo, Fabricio Lima Brasil, Allison Candido Lima Santos, Luzenildo de Sousa Batista Junior, Savio Pereira Fonseca Dutra, Carlos Eduardo Coelho Freire Batista

**Affiliations:** ^1^Digital Video Applications Lab, Universidade Federal da Paraíba, R. dos Escoteiros s/n, Mangabeira, 58055-000 João Pessoa, PB, Brazil; ^2^Santos Dumont Institute, Edmond and Lily Safra International Institute of Neuroscience, Rod. RN 160, Km 03, No. 3003, 59280-000 Macaiba, RN, Brazil

## Abstract

Deafness, an issue that affects millions of people around the globe, is manifested in different intensities and related to many causes. This impairment negatively affects different aspects of the social life of the deaf people, and music-centered situations (concerts, religious events, etc.) are obviously not inviting for them. The Auris System was conceived to provide the musical experimentation for people who have some type of hearing loss. This system is able to extract musical information from audio and create a representation for music pieces using different stimuli, a new media format to be interpreted by other senses than the hearing. In addition, the system defines a testing methodology based on a noninvasive brain activity recording using an electroencephalographic (EEG) device. The results of the tests are being used to better understand the human musical cognition, in order to improve the accuracy of the Auris musical representation.

## 1. Introduction

Deafness is an issue that affects a significant part of the population in all countries, but the incidence increases especially in the developing ones, like Brazil, in which deafness is defined as bilateral hearing loss of forty-one decibels [1]. According to the Brazilian Institute of Geography and Statistics (IBGE) census, 5.10% of Brazilian population have some type of hearing impairment [2]. On a worldwide scale, according to the World Health Organization (WHO) [3], the number of people who have disabling hearing loss is around 360 million, and 1.1 billion young people (aged between 12 and 35 years) are at risk of hearing loss due to exposure to noise in recreational settings. It is important to point out that hearing loss impacts not only leisure's problems, but also all kinds of daily activities. These data reinforce the need for solutions to reintegrate this group in activities which are centered in auditory stimuli, such as musical events.

Different solutions are being developed in order to help people with hearing disabilities to consume music. The Model Human Cochlea [4] is an example of device for musical accessibility, which uses a musical representation in a vibrotactile display. The vibration is expressed through eight voice coils embedded in the back of a chair and distributed in a four by two matrix. Each voice row is associated with a different channel and represents a specific element of the music.

Another project uses a similar approach, but using different tools to represent vibrations. The Haptic Chair [5] is responsible for vibrations transmissions through contact speakers (transducers). The main concept used in this device is to enhance music vibration without any artificial effect; in other words, it amplifies the pure audio signal. Such approach allows the subjects with partial deafness to hear the audio transmitted through transducers vibrations. In addition, a visual display is used to expose visual sequences created based on the music, allowing the subjects to follow information through elements such as shapes, with different brightness and colors. The system displays visual effects that match the current note duration, pitch, and also loudness, timbre, and key changes. Different configurations were tested using music, visual display, and the Haptic Chair— the test results, obtained through the conduction of usability questionnaires, indicated that the difference between the Flow State Scale score of the music with Haptic Chair and music with visual display and Haptic Chair configurations was not significant. When the participants were asked about the configuration that represents a first choice, 54% preferred consuming music with Haptic Chair only and 46% preferred consuming music with the visual interface and the Haptic Chair.

The use of visual interfaces for deaf people is shown to be important, because it enhances the possibility of immersion and, together with the haptics information, increases the amount of information for the music representation. A study using fMRI [6] indicated that some visual stimuli are able to activate the auditory cortex in deaf people, in the same way it happens when people with no hearing impairment listen to sound. This study suggests the relevance of visual information for the comprehension of interaction of deaf people with different forms of musical representation, which means that video may be used to help deaf people to understand and consume music.

Brain-machine interfaces (BMI) allow direct translation of electric or metabolic brain activity into control signals of external devices or computers bypassing the peripheral nervous and muscular system. The neural or metabolic brain activity can be recorded from sensors outside the brain such as using electro- or magnetoencephalography (EEG/MEG), functional magnetic resonance imaging (fMRI), or near-infrared spectroscopy (NIRS) [7].

The aim of the current study is to translate audio stimuli to a new media composed of filtered audio and tactile vibrations, in order to verify whether this combination can be used to overcome deafness for the perception of music and also understand how the brain interprets these new tactile information. The testing is based on information captured from the subjects using inexpensive commercially available EEG electrodes. The system named Auris [8] is responsible for reproducing the new media created from the original audio. The reproduction is done through two different devices, one named Auris Chair and another named Auris Bracelet. The union of these two devices is responsible for reproducing musical elements, such as melody, rhythm, and harmony.

## 2. Materials and Methods

The initial discussions for the Auris System development arose from the restrictions faced by the VLibras project [9], which developed solutions aiming at people with hearing impairment. Musical information was not studied by this project, and this gap motivated the development of the Auris System. While researching the state of the art, we understood that most of the previously presented approaches were trying to translate audio into some other media that could be perceived by people with hearing impairment, but most of their results and analysis were guided by subjective information, which could lead to imprecise conclusions.

The following subsections, thus, aims to explain how the Auris System was conceived, how it works, the methodology to evaluate how effective the system is, and how similar, from a neurological standpoint, is hearing music, compared to the experience provided by Auris System.

### 2.1. Auris System

The Auris System was designed to foster the possibility of a better musical experience for the hearing impaired population. The system brings a set of tools that converts audio to a new media (consisting in a filtered audio synchronized with tactile impulses) in order to be played through loudspeakers and special haptic interfaces, improving the musical experience for deaf people, or anyone with hear deficits. The system is currently composed of the Auris Chair, the Auris Bracelet, and two integrated software components: (a) the Auris Core and (b) the Auris Controller, whose development was deeper described in a previous study [8]. Some software components were further improved and are explained in this work.

The Auris Chair is composed of four six-inch speakers positioned on the back of the Auris Chair and a subwoofer positioned on the seat, both acoustically insulated. This is the component responsible for playing back the filtered audio (Auris Audio). The Auris Bracelet consists of a series of vibration motors attached to small plastic plates disposed in an 1 × 6 matrix. The Auris Bracelet function is to represent harmonic or melodic information present in the songs through different vibration patterns and can act with different configurations in relation to the combination of motors vibration and represented musical tones.

**Figure 1:**
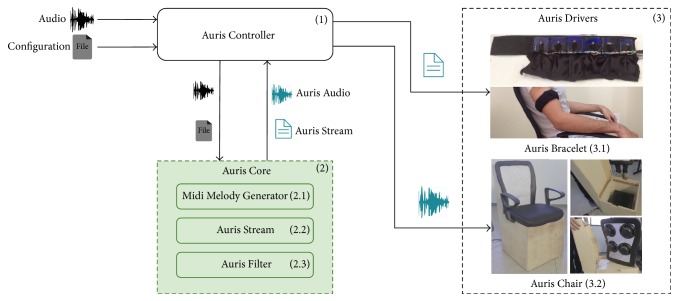
Schematic overview of the Auris System.

The System architecture is depicted in Figure 1 describing the following components: Auris Controller; Auris Core, Midi Melody Generator, Auris Stream, Auris Filter, Auris Drivers, Auris Chair, and Auris Bracelet.

The Auris Controller (1) is responsible for managing the functioning of the other components, in order to provide the conversion between regular audio in a media that is perceivable by deaf people. The Auris Controller starts the process by receiving two inputs: an audio file (*.wav* or *.mp3*) and a configuration file. The Auris Core (2) receives the audio and configuration files, processes them with its subcomponents (Midi Melody Generator, Auris Stream, and Auris Filter), and returns the generated artifacts. The audio process for generating the Auris Stream and Auris Audio artifacts, consists, respectively, in extraction of the melodic information from the original audio file using the implementation made by [10], which provides a MIDI representation of it through MELODIA algorithm developed by [11]; after that, the extraction of specific information from MIDI file, used to compose the Auris Stream file, can be executed; for the Auris Audio, the required process consists in generating a filtered version of the audio, in order to amplify the low level frequencies that can also be perceived by the tactile system. Those two processes are required for generating the artifacts, which will be forwarded to the Auris Controller, responsible for commands of the Auris Chair (3.1) and the Auris Bracelet (3.2) (via its drivers) to play filtered audio and the extracted melody information, according to the defined configuration.

The methodology used for the melodic musical information extraction was developed by [11] and divided into four steps. In the first one, the experimenter utilizes a sinusoidal extraction process to obtain the spectral peaks, which serve as input for the salience function. In the second step, he gets the possible melody candidates on fundamental frequency. The third step creates the pitch salience, and then verifies which pitch belongs to the original melody. In the last step, from the pitches belonging to the melody, a selection occurs generating the melody in fundamental frequency.

After the extraction step previously explained, it is possible to access information regarding the notes distribution in time (moment that a note starts and ends) from the melody in the audio and then describe it based on our configuration file. The configuration file is responsible for specifying and providing the motors and the notes represented by them; the vibration range of each motor; the notes distribution in bracelet; and the method of representation. Finally the Auris Stream file can be built as an archive which contains the information of the extracted music and instructions of how we are going to transmit melody though the Auris Bracelet. The Auris Stream file enables the visualization of which motor will vibrate by using his own Identifier (ID), the start/end time of vibration which is measured in milliseconds, and the intensity which can range from 0 to 255, where 0 is the minimum and 255 is the maximum range.

#### 2.1.1. Musical Representation

This section aims to clarify relevant aspects regarding the way Auris presents music to its users. As previously mentioned, the Auris System converts regular audio containing music to a filtered version, which is combined with a MIDI file, in order to be reproduced by the loudspeakers attached to the chair and also by the haptics interface.

More intense and strong vibrations are more relevant to the users perception than weak ones. The purpose of Auris Filter is to enhance weak vibrations using an application to increase the gain of interest frequencies. The vibration frequencies are sensible to touch and vary from 10 Hz to 1000 Hz [12]. Vibration frequencies above this range are very difficult to be perceptible to touch and the range from 200 Hz to 300 Hz is the best touch sensitive frequencies [13].

**Figure 2:**
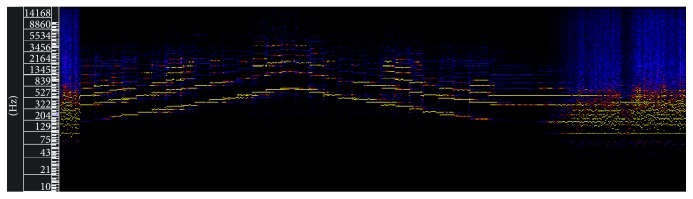
Violin notes scale, generated by Sonic Visualiser.

These frequencies ranges are not adequate considering the fact that music will be carried by the audio to be filtered. In other words, it can not represent most of the music timbres, rhythm variations, and other different musical elements. This representation problem can be easily noticed through the violin scale spectrogram generated by Sonic Visualiser [14] application and presented in Figure 2.

In Figure 2, we have a crescent and decrescent violin notes scale sequence. The red, yellow, and blue colors represent, respectively, the high, moderate, and weak intensity of the signal, in that spectral region. It is noticeable that notes above 3 kHz are more present.

For this task, we used the Essentia library [15] and Pure Data Platform [16]. Essentia is used when audio filter and gain configuration are the same for the whole song and when it is desired to use the maximum processing speed. Pure Data is used when it is necessary to process the song in real time, allowing the experimenter to change the parameters such as filter configuration, cutting frequency, gain, or bandwidth (Bandpass filter) also in real time while the audio is processed. This approach allows the use of Pure Data not only for music, but also for ambient sound if a microphone is connected into the computer.

According to the hardware configuration used to build the Auris Chair, the capacity of frequency representation ranges from 70 Hz to 20 kHz on speakers, and 20 Hz to 200 Hz on Subwoofer. The song “Cleanin' Out My Closet” by Eminem was arbitrarily chosen and used during our tests and will be taken as an example in order to highlight our decisions regarding complementing the audio with a haptics interface. This particular song has a very strong presence of frequencies below 5000 Hz, since Hip hop is a genre that commonly provides music with low frequencies. The limit for vibration frequencies sensible to touch ranges from 10 Hz to 1000 Hz. For this song, in this context, if we do not use the Auris Bracelet and use only the filtered sound representation, the range from 1 kHz to 5 kHz would not be well represented. Since the Auris Bracelet makes a translation from musical frequencies into tactile vibrations, including frequencies that would not be perceivable through the filtered audio, it facilitates the process of identifying a larger part of the musical elements from what is playing, even if the frequencies are above 1 kHz.

### 2.2. Methods

Different approaches were used in order to evaluate subject perceptions. In order to obtain the most expressive feedback from the subjects involved, among deaf and hearing persons, two different processes were used during the development of the Auris System. The first approach has been through the usability questionnaires, but with drawback of subjective answers by the participants. In the second one, we used an electroencephalogram (EEG) test to obtain a measured feedback from participants. To acquire the electrical activity data in this test, we used an Emotiv EPOC® [17]. This device was used for recording brain signals from the participants during the system experimentation. Deaf and hearing participants had their data collected during part of the experiments.

In all experiments, the participants were informed about the process that would happen and had the freedom to choose the music volume that would be played. In the first test approach, the participants were not isolated and could watch the other participants' experiments. In the second, with the acquisition of EEG signals, participants were isolated in an enclosed environment in order to avoid interfering variables.

Both groups of participants, between deaf and hearing persons, had just had contact with the test evaluator, but, in the deaf group, the participation of a professional interpreter of the Brazilian sign language was necessary. Distinct artifacts were chosen for integrating the test scenarios, and they were used in different configurations, depending on the group which the test was being applied to. These artifacts were the Auris Chair, Auris Bracelet, a video monitor, and an in-ear headphone.

#### 2.2.1. The Auris System and the Visual Stimuli Comprehension

In order to understand the results presented by the author [5], where the visual interface was not significant together with the Haptic Chair, we decided to use a visual element in the first step on our system evaluation. This visual interface has the role of displaying the public video used in our tests. The video contents are composed of a classical dance, more precisely a waltz, where two persons dance according to this music style. The objective here was to evaluate the comprehension by the deaf participants between the audio vibrations and the displayed video content. For this purpose, the video was combined with three different music pieces from different genders.

The songs chosen were

- Eminem, Cleanin' Out My Closet
- System Of A Down, B.Y.O.B.
- Tchaikovsky, Waltz of the Flowers.

The experiment was applied at the institution Fundação Centro Integrado de Apoio à Pessoa com Deficiência (FUNAD). The deaf participants were invited to sit on the Auris Chair and feel, or experience, the three different music pieces. Each music piece was played separately but combined with the same video. The participant was informed about every music change, and only after the three executions were they asked about which song most matches the video presented.

#### 2.2.2. The Auris System and Electroencephalogram Tests

The second approach was ran with acquisition of EEG signals. In this way, deaf and hearing participants had their electrical brain activity recorded using an Emotiv device. The acquired data were captured during four music types' executions. Each participant listened to or felt one musical sample of each tag used in the experiment, according to Table 1.

Different music pieces were chosen for composing this part of tests, more specifically eight. These songs were classified into four different types, defined according to emotional tags, informed by different users around the world in a crowdsourcing technique, as proposed by [18]. After that, the eight different music pieces could be separated in pairs, according to the different tags. These songs and types can be better observed in Table 1.

Brain activity data acquisition from participants was ran at Universidade Federal da Paraíba (UFPB). The Auris System was used as well as the tactile devices, and the deaf subjects were invited to sit in an Auris Chair and/or use the Auris Bracelet to feel the music vibrations. In the case of hearing persons, they used an in-ear headphone for listening to the music pieces.

#### 2.2.3. Experiment Setup

In the two sessions of experiments, we had a total of 13 participants (deaf or hearing person); their ages and devices used on each test can be found on Table 2.

The pure audio signal was used in both tests, although the Auris Filter provides different configurations of gain and filters; it was necessary to evaluate the feedbacks provided by the participants, before applying any modification on audio signal. In addition, participants that used the Auris Bracelet had just had contact with one configuration of the Auris Bracelet (the most simple), in which each motor vibrates on the same frequency and alternates the vibrating motor for melody representation.

Participants that used the Emotiv device had their data recorded using the Test Bench version 1.0.0.0 software provided by Emotiv SDK. For offline analysis we used EEGLAB version 13.5.4b, importing the.edf file containing the data acquired from each volunteer, using a BIOSIG load option. Channels presented in the Emotiv interface were defined, as well as their specific scalp locations. Before plotting the data, the baseline from the sample was removed and the Independent Component Analysis (ICA) decomposition was executed.

After preprocessing the data, it was possible to plot the graph spectra and maps, for analyzing a component contribution at one specific channel and power. In our case, the channel F3 and the frequency 12 Hz were specified to reproduce the graphs from 100 percent of the acquired data.

Previous work has shown that mental imagination of sound generally elicits an increase of alpha band activity (8–12 Hz) in the electroencephalogram (EEG) [19]. Based on that, we chose 12 Hz in our data analysis.

## 3. Results

Experiments were conducted in order to evaluate the Auris System. In the first session of experiments we evaluated the audio-video correlation by the deaf participants, using the Auris System. The evaluation consisted of usability questionnaires. Afterwards we conducted experiments that analyzed the deaf and hearing patients feedback, through the EEG signals acquired with the Emotiv device; the experiment aims to evaluate the participants experience with quantitative data, providing basis for establishing the differences between deaf and listeners perception.

### 3.1. Questionnaire Results

**Figure 3:**
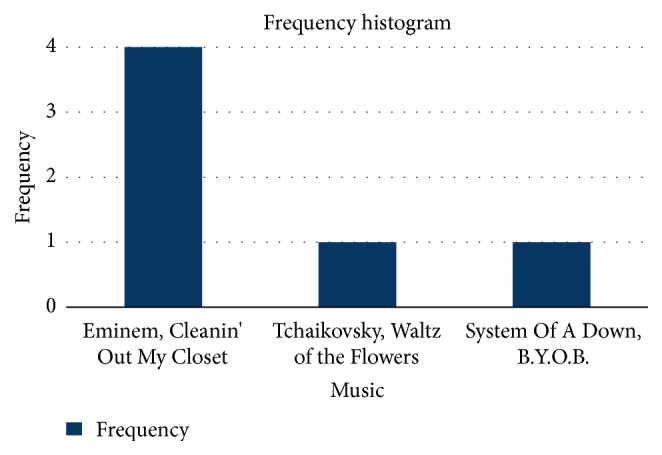
Association between music and video by deaf participants.

The first session of experiment evaluated the Auris System with the support of visual stimuli, in order to facilitate the comprehension of music. The users were exposed to three different songs as soundtrack to a single video instance. The participants were asked which of the songs was best related to the video. We were able to produce a frequency histogram analyzing the participants answers, which are represented in Figure 3. The graphic expresses on the *x*-axis the songs listened to by the participants and the *y*-axis expresses the number of participants that chose that song with the video.

The original audio from the video content was the classical song by* Tchaikovsky, Waltz of the Flowers*. According to the graphic it is possible to observe that most participants have chosen the song by* Eminem, Cleanin' Out My Closet*. Despite the fact that these two songs have different styles, they have similar tempo information. This common characteristic was evidenced through hardware configurations of the Auris Chair, which, for the test, was composed of speakers and a loudspeaker component (subwoofer), that emitted filtered audio, so the rhythmic information had a significant presence in the music translation reproduced through the Auris Chair.

Two different musical software programs were used to extract specific musical aspects such as tempo, energy, and key results from the used music. The software programs used were Ableton Live 9 [20] and Mixed in Key 8 [21]. After this extraction, we were able to construct Table 3.

Analyzing Table 3 we can perceive the similarity present between* Eminem, Cleanin' Out My Closet,* and* Tchaikovsky, Waltz of the Flowers,* songs. Both songs express a similar tempo and energy information, indicating that the Auris Chair improves the rhythmical, dynamic, and volume information from the audio input, according to the FUNAD test participant's answers.

Based on this scenario, we are able to foresee that the possibility of audio-video association for deaf participants can be enhanced by the use of the Auris System. Four different profoundly deaf participants (born deaf) were able to associate the song with the same tempo (“Cleaning out my closet”) with the video. The most precise association between audio and video was made by one subject that was not deaf from birth, choosing* Tchaikovsky, Waltz of the Flowers*. One participant, which is profoundly deaf from birth, associated the* System Of A Down, B.Y.O.B.,* music correctly.

The contact with the subjects and the stories and experiences that they brought to our attention were as rich as the data produced by these experiments. Most of those experiences were regarding their relationship with situations that were audio centered or in which audio played an important role (such as attending music concerts and playing video games) and their workarounds to improve such experiences (getting closer to the loudspeakers, using headphones and/or loudspeakers touching the neck or palms of the feet). Many different rounds and questionnaire test versions were conducted until we were able to have sufficient amount of data to analyze.

From those experiments we could empirically detect that some subjects became more sensible to music through the usage of the Auris System, and considering subjects with the same profoundness of deafness, the distinguishing factor was related to the subjects past activities and relationship with audio centered situations. One particular subject highlighted such suspicion: although he was born with profound deafness, he was able to distinguish most musical characteristics, even being able to relate genres and similar bands—during the interviews he stated that, during his whole life, he enjoyed attending music concerts and standing close to the loudspeakers. The other subjects, with the same level of deafness and which did not demonstrate a high level of musical sensitivity, all expressed in the interviews that they did not have a history of attending audio centered events. Some even mentioned that they felt socially excluded of such events.

In order to better understand the experience provided by the Auris System and attenuate the amount of subjectivity on the data that we were considering for our analysis and in order to have more precise directions to identify which elements of the system should be modified to produce a better musical representation using other media than audio (e.g., what parameters shall be considered for the audio filtering and what tactile representation suits better this type of harmony or melody), the next subsection discusses our efforts in such direction, where EEG data collection was used for the experiments.

### 3.2. EEG Results

For ease of presentation and hence understanding, all analyses were focused on data from the frontal site in the left hemisphere, on channel F3. This channel was selected because it is usually used in studies involving auditory responses [22] and it is also included in vibrotactile discrimination loop [23]. In order to know which components contribute most strongly to which frequencies in the data, we plotted the mean log spectrum of a set of data epochs at all channels as a bundle of traces. For 12 Hz we plotted the relative topographic distribution of power (Table 4).

For construction of Table 4 the EEG data collected by the deaf and hearing volunteers was used, which have listened to/felt the same music tag, defined as energetic and positive, according to Table 1.

Table 4 shows that similar components contribute strongly to deaf participants, and other similar components contribute strongly do hearing persons. Deaf participants showed also a higher power for lower frequencies, what was not seen in the hearing participants.

## 4. Discussion

This study evaluated the functioning and translation of audio stimulus from music pieces to a different representation using low frequency sound and tactile vibration in deaf and hearing volunteers. The evaluation considered the recording of brain signal with noninvasive EEG electrodes (using the Emotiv device) while participants consumed music; the deaf participants used the Auris System, composed of the drivers Auris Chair and the Auris Bracelet.

The first results suggest that deaf patients that never had a similar experience related to music were capable of identifying and associating musical information concerning the rhythm and energy present in the music, associating them accordingly with a video presented to them, with the exception of one participant that was not deaf from birth. The audio-video association made by the deaf participants was possible through the use of the Auris System and the Auris Chair hardware.

Based on the previous findings and relating them to the results presented by [5], in which deaf participants express an nonsignificant effect after addition of the visuals element combined with the Haptic Chair, we realized that the problem is probably not related to the visual and tactile feedback association, suggesting the necessity of more depth studies related to visual representation for music aimed at deaf people.

During the period of research development and survey of the related works, we noticed that the previous findings [4, 5] associated with our context presented their results based on less objective evaluation mechanisms—the type of information that can be obtained through EEG may express more. The state of the art presents findings in which the participants provided the data through usability questionnaires, expressing the strength of emotions and perceptions and explaining such information to a professional interpreter. These evaluation methodologies were used in the first step of our analysis, but a discomfort was perceived in the participants, in addition to a difficulty in describing their perceptions. Based on this and in other previous findings, which use EEG signals as part of the evaluation [11, 24], the necessity and the possibility for a more expressive feedback are perceivable, exposed in the second session's results.

With use of EEG, recent studies [25] recorded the mismatch negativity of the auditory event-related potential to changes in musical features in adolescent cochlear implants users and in normal-hearing age mates. They reported that behavioral discrimination of rhythm and melodic contour may be significantly improved, even from short term training, whereas detection of changes in pitch was poor and unaffected by music training.

Music interpretation was a hard task for some deaf participants. We believe that, despite the translation of music elements (rhythm, harmony, melody, and timbres), patients had no previous experience to these vibrations patterns and, even though they maybe perceiving, they could not differentiate important elements of the songs. We believe that continuous musical stimulation using different media other than audio during daily life activities would support a natural learning curve for a pattern recognition that enhances music experimentation.

Further studies are necessary to clarify brain dynamics associated with vibrotactile and bass-sound impact in deaf people. Such understanding will guide the development of a better musical representation that is not any translation, but one that might lead to experiences similar to those experimented by hearing people.

## 5. Conclusion

The presented results are encouraging, but there is still a long way to develop a system that significantly helps people with some degree of deafness to achieve a satisfactory level of musical perception. Nevertheless, the learning resulting from conducting the tests exceeded our expectations. Several reports and empirical conclusions from the tested subjects were also primordial for the accomplishment of this work. The need to improve the representation system is evident and urgent, so users can easily identify the different elements involved in music (rhythm, harmony, melody, and timbres) and offer better ways of representing and expressing these elements (visual stimuli, more levels of tactile stimuli, etc.); it is clear that the methodology evaluation has also to consider a broader universe of subjects and particularities involved in musical perception, such as cultural context (e.g., different cultures use different musical scales and focus on different elements and thus people from different places may perceive music differently).

The evaluation methodology was based mainly on the interpretation of EEG data; thus a more in-depth interpretation of the new data generated on the forthcoming tests is required and also a bigger universe of test to analyze. Those data should be used to better understand how the musical cognition happens by hearing persons and to produce a better understanding of what the Auris System's users are experimenting, so we will have better possibilities to bring one experience closest to the other.

The Auris representation system (currently consolidated in the chair) is being redesigned in two versions: one with miniaturized components such as mobile/wearable option and another to coexist in environments where sound is consumed by hearing people. An integration with the VLibras system [9] is under development, which will make VLibras able to represent musical information by offering a visual representation of musical elements—when used with the Auris system, the avatar of the VLibras system presents the lyrics of the song in LIBRAS (Brazilian sign language), moving the trunk according to the rhythm and emphasizing moments of apex. Other visual references representing musical elements are being studied to be used along with the VLibras avatar.

Our preliminary results reinforce findings of [26], where vibrotactile channel functions as a valuable feedback modality in a BMI-controlled setting. With improvements of stimulation paradigm, training protocol, and device functioning we believe that people with different levels of hearing impairment will be able to use this music translation device in their daily life activities in the future.

## Figures and Tables

**Table 1:** Table of the music and tags used on tests.

Music type *(tags)*	Artist, title
Calm positive	Mike Oldfield, Harmonia Mundi
	Pink Martini, White Christmas
Energetic positive	George Benson, All Of Me
	Jennifer Lopez, Let's Get Loud
Dark calm	David Sylvian, Bringing Down The Light
	Matanza, Clube dos Canalhas
Dark energetic	Celine Dion, Regarde moi
	Placebo, Meds

**Table 2:** Table of participants information and characteristics of the tests.

Test	Participant	Type	Age	Used devices		
				Auris Chair	Auris Bracelet	EEG
FUNAD	1	Deaf	15	X		
	2	Deaf	27	X		
	3	Deaf	28	X		
	4	Deaf	31	X		
	5	Deaf	46	X		
	6	Deaf	62	X		

UFPB	2	Deaf	27	X		X
	7	Deaf	16	X		X
	8	Deaf	26	X		X
	9	Deaf	28	X	X	X
	10	Deaf	29	X	X	X
	11	Hearing person	20			X
	12	Hearing person	23			X
	13	Hearing person	23			X

**Table 3:** Table of musical analysis.

Music	Tempo (bpm)	Energy	Key result
Tchaikovsky, Waltz of the Flowers	141.88	3	D
Eminem, Cleanin' Out My Closet	147.99	4	Am
System Of A Down, B.Y.O.B.	97.23	7	G#m

**Table 4:** Table of relative topographic distribution of power, from representative deaf (1a and 2a), and hearing participants (1b and 2b) for 12 Hz at channel F3. Each colored line represents the spectrum activity of one single data channel.

Nr.	a	b
1	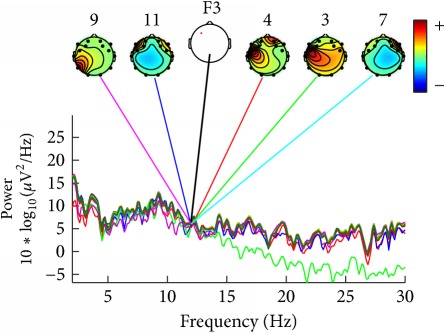	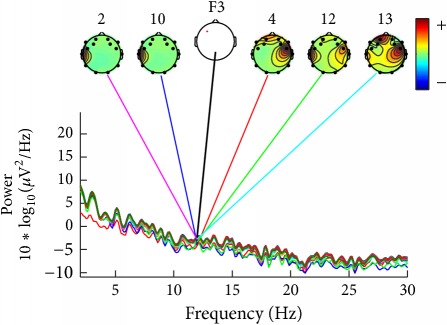

2